# Microbiological Safety of Selected Albanian Foods in Relation to EU Criteria: Evidence from Artisanal Dairy and Meat Supply Chains

**DOI:** 10.3390/foods15132335

**Published:** 2026-07-01

**Authors:** Rozeta Hasalliu, Arbnora Durmishaj, Pajtim Bytyçi, Klotilda Sula, Aida Shkurti, Edison Axhami, Ylber Karanxha, Klejda Furriku, Małgorzata Ziarno

**Affiliations:** 1 Department of Food Science and Biotechnology, Faculty of Food Biotechnology, Agricultural University of Tirana, Rruga “Pajsi Vodica”, Kodër-Kamëz, 1029 Tirana, Albaniapajtim.bytyqi@ubt-uni.net (P.B.); ksula@ubt.edu.al (K.S.); ashkurti@ubt.edu.al (A.S.); edisonaxhami45@gmail.com (E.A.); 2 Department of Marketing and Engineering, Faculty of Business and Justice, Alexander Xhuvani University of Elbasan, Rruga “Ismail Zyma”, 3000 Elbasan, Albania; 3 Department of Food Science and Biotechnology, Faculty of Food Biotechnology, Albanian Food Authority, Rruga “Pajsi Vodica”, Kodër-Kamëz, 1029 Tirana, Albania; 4 Department of Food Technology and Assessment, Institute of Food Science, Warsaw University of Life Sciences—SGGW (WULS–SGGW), Nowoursynowska 159c St., 02-776 Warsaw, Poland

**Keywords:** artisanal cheese, raw milk hygiene, food safety, ISO standards, microbial risk, *Salmonella* spp., *Listeria monocytogenes*

## Abstract

Published microbiological data for Albanian artisanal dairy and meat supply chains remain limited, particularly for origin-specific cheeses, raw farm milk, and selected meat products. This targeted exploratory study examined 145 physical samples representing independent product lots or, for raw milk, independent bulk-milk lots obtained in separate collection events. The samples were organised into 29 organism-by-matrix analytical categories, with five independent experimental units in each category. ISO-based methods were used to enumerate hygiene indicators and screen for selected pathogens. Beta-glucuronidase-positive *Escherichia coli* counts were below 10 CFU/g in cow milk feta-type brined cheese and sausage, while Enterobacteriaceae counts were below 10 CFU/g in goat yoghurt and pistachio cake. In three raw-milk cheese-origin groups with quantified *Escherichia coli* data, mean counts ranged from 3.08 to 3.25 log CFU/g. For Liqenasi and Bardhoke, all five results in each category were >300 CFU/g and were treated as right-censored; the exact counts above this value were not quantified, so these observations were excluded from numerical summaries. Raw milk aerobic colony counts ranged from 4.52 to 6.52 log CFU/mL. Unconfirmed presumptive *Salmonella* spp. culture screening findings were obtained for 5/5 frozen chicken portions tested at 10 g and 5/5 pork portions tested at 25 g. *Listeria monocytogenes* was not detected in 5/5 ready-to-eat grilled chicken portions, each 25 g. Findings apply only to the sampled lots and support confirmatory sampling, complete lot documentation, and quantitative follow-up of right-censored results.

## 1. Introduction

Microbiological safety in short, regional and artisanal food supply chains is a public health issue with relevance beyond national borders. Dairy and meat products produced under small-scale conditions may retain cultural and sensory value, but they can also present microbiological risks when milking hygiene, cooling, heat treatment, manual handling or retail storage are not consistently controlled. Indicator organisms, such as *Escherichia coli* and Enterobacteriaceae, are useful for evaluating process hygiene and post-processing contamination in defined food categories. In contrast, pathogens such as *Salmonella* spp. and *Listeria monocytogenes* are interpreted primarily in relation to food safety criteria, where applicable. For coagulase-positive staphylococci, including *Staphylococcus aureus*, risk interpretation requires caution because food safety assessment is linked to staphylococcal enterotoxins, whereas bacterial counts alone provide mainly process hygiene information.

Albania is a relevant case for this problem because small-scale dairy and meat supply chains coexist with progressive alignment to EU food safety expectations. Traditional goat and sheep cheeses are widely consumed, and form part of local food identity, yet structured published data on their microbiological quality remain limited [1,2]. Some cheeses reach consumers as raw or ripened products, produced from pasteurised or raw milk and under variable sanitary conditions. Their safety and hygiene status are shaped by the quality of incoming milk, heat treatment where used, equipment and surface sanitation, starter culture performance, curd handling, ripening conditions and storage temperature.

The evidence gap is not only geographical. It concerns specific matrices, microorganisms, production stages and regulatory questions. For Albanian cheeses, available data do not sufficiently distinguish origin, milk status, ripening or brining conditions and the sampling stage at which EU process hygiene criteria could apply. For raw milk, farm-level total aerobic colony counts need to be interpreted against the EU rolling geometric average framework, not as single-sampling compliance decisions. For retail meats, *Salmonella* screening must be linked to the test portion, product category and the absence of serotyping. For ready-to-eat products, especially cooked poultry, *Listeria monocytogenes* results require information on ready-to-eat status, shelf-life stage and growth potential. This study was designed to address these gaps at the screening level by including named cheese origins, raw milk from three farms and selected retail meat and ready-to-eat products, while keeping food safety endpoints separate from process hygiene endpoints.

European food law places the protection of human health at the centre of food-chain regulation. Microbiological criteria support the assessment of food safety and process hygiene under Regulation (EC) No 178/2002 [3] and Commission Regulation (EC) No 2073/2005 [4], while specific hygiene requirements for food of animal origin, including microbiological requirements for raw milk, are laid down in Regulation (EC) No 853/2004 [5]. Commission Regulation (EU) 2024/2895 amended the *Listeria monocytogenes* criterion for ready-to-eat foods able to support growth and applies from 1 July 2026 [6]. A clear distinction among food safety criteria, process hygiene criteria, and screening benchmarks is necessary to avoid overinterpretation.

Albania, as an EU candidate country, is progressively aligning its food safety legislation with the EU acquis. In the present study, EU microbiological criteria were used as interpretive benchmarks unless direct applicability to a given Albanian product category and sampling stage could be established. This approach allows the results to be discussed in relation to EU-aligned control priorities, without presenting the study as formal enforcement sampling. Previous studies on artisanal dairy products in Europe have reported variable hygiene performance, reflecting differences in raw milk quality, environmental contamination, producer training and standardisation of manufacturing steps [7,8,9,10,11,12,13,14]. Variability in raw milk quality between farms has also been described in Mediterranean production systems, where total aerobic colony counts are influenced by milking hygiene, cooling rate and storage temperature [15]. In Eastern and South-Eastern Europe, comparable datasets remain less abundant than in long-established EU monitoring systems, especially when raw data, sampling stage and criterion applicability are considered together.

The study addressed four research questions. First, did the culture-screening procedures yield unconfirmed presumptive *Salmonella* spp. findings in the tested raw-meat portions, and was *Listeria monocytogenes* detected in the ready-to-eat grilled chicken portions? Second, how did process hygiene indicators vary among product groups, sampled cheese-origin groups and raw milk farms? Third, how did the results compare with EU food safety or process hygiene criteria when the product status, sampling stage and test portion allowed such a comparison? Fourth, which missing data prevent formal compliance classification and define the needs for follow-up surveillance?

## 2. Materials and Methods

### 2.1. Sample Collection

The study used a targeted exploratory sampling design intended to compare selected matrices and production stages and to identify microbiological signals, not to estimate national prevalence. The experimental unit was one independently produced product lot represented by one physical sample submitted for one microbiological endpoint. For raw milk, the experimental unit was one independently collected bulk-milk lot from a separate collection event. Each of the 29 organism-by-matrix analytical categories comprised five independent experimental units, yielding 145 physical samples. Repeated product or origin names in different categories represented separate five-lot sets collected for different microbiological endpoints. No physical sample was reused across analytical categories. Lot identifiers and producer identities are not reported to protect commercial confidentiality.

Samples were collected from markets, farms or production sites in Albania during 2025–2026. Cheese samples used for the process-hygiene assessment were collected at production sites after manufacture. Supplementary Table S3 lists all 29 analytical categories, origin or anonymised source, collection-site type, collection period, experimental-unit type, basis for independence, storage status and ready-to-eat status. Lot codes and producer names are omitted. Where the same product or origin appears under more than one endpoint, each row represents a separately collected set of five independent product lots.

The sampling plan included traditional goat and sheep cheeses from named origins, cow-milk feta-type brined cheese, goat yoghurt, frozen chicken fillet, grilled chicken fillet, pork fillet, sausage, pistachio cake, and raw cow milk from three farms. Samples were transported under conditions appropriate to their product status. Frozen chicken was transported at −18 °C, while chilled products were transported at approximately 4–8 °C. Samples were analysed within 24 h of receipt.

Available product-characterisation data are summarised in Supplementary Table S4. The table reports milk or thermal status, ripening and brining conditions where recorded, packaging, storage temperature, shelf life and intended use. The grilled chicken fillet was a cooked, ready-to-eat product with a declared shelf life of 14 days. The sausage was a cooked, ready-to-eat product. pH and water activity were not measured in the analysed samples; literature-derived values previously shown in the table were removed and were not used to assess growth potential or regulatory applicability.

### 2.2. Microbiological Analyses

Microbiological examinations used culture-based procedures documented in the retained laboratory records and linked to the cited ISO reference methods. Supplementary Table S1 reports only conditions verified in those records, including the documented test portion, preparation steps, media, incubation conditions, result type and reporting limit.

For enumeration, beta-glucuronidase-positive *Escherichia coli* was determined according to ISO 16649-2:2001 [16], Enterobacteriaceae according to ISO 21528-2:2017 [17], coagulase-positive staphylococci according to ISO 6888-2:2021/Amd 1:2023 [18], and total aerobic colony counts at 30 °C according to ISO 4833-1:2013/Amd 1:2022 [19]. Results were expressed as CFU/g or CFU/mL. The usual LOQ for the enumerative methods was 10 CFU/g or 10 CFU/mL unless a different dilution or plating scheme applied.

Screening for *Salmonella* spp. used an ISO 6579-1-based culture workflow comprising pre-enrichment, selective enrichment, selective plating and basic biochemical screening [20]. The outcome is reported throughout as an unconfirmed presumptive *Salmonella* spp. culture-screening finding. This designation was assigned when colonies with typical growth characteristics on selective media were supported by the basic biochemical reactions documented in the laboratory. Complete confirmatory identification, serological agglutination, species-level identification, serotyping and molecular characterisation were not performed. Detection of *Listeria monocytogenes* was performed in accordance with ISO 11290-1:2017 [21]. The test portion was 10 g for frozen chicken fillet, 25 g for pork fillet and 25 g for grilled chicken fillet. Because the frozen chicken test portion was 10 g, the finding was not interpreted against an EU absence-in-25 g criterion. Coagulase-positive staphylococci were enumerated in accordance with ISO 6888-2, and results below the quantification limit were reported as <10 CFU/g. Staphylococcal enterotoxins were not analysed.

The organism-by-matrix testing plan is presented in Supplementary Table S5. It distinguishes food safety endpoints from process hygiene endpoints and explains why certain organism–matrix combinations were not tested. The ready-to-eat cheeses were included for process-hygiene comparison only. *Salmonella* spp. and *Listeria monocytogenes* were not tested because the available analytical programme was limited to indicator organisms and coagulase-positive staphylococci. Consequently, no direct food-safety conclusion can be drawn for these cheeses.

Coliforms were enumerated in accordance with ISO 4832:2006 [22]. Results below the quantification limit were reported as <10 CFU/g. For Escherichia coli, coliform, and total aerobic colony-count categories reported as >300 CFU/g, 1.0 mL of the 1:10 initial suspension corresponded to 0.1 g of sample. A plate record of more than 30 colonies, therefore, corresponded to >30 CFU/0.1 g, or >300 CFU/g. Exact counts above this reporting category were not retained, and the observations were treated as right-censored. The discussion therefore focuses on *Escherichia coli* and Enterobacteriaceae as the indicators most directly related to the EU microbiological framework [4].

### 2.3. Statistical Analysis

Quantitative microbiological results were presented as CFU/g or CFU/mL and, where appropriate, as log10 CFU/g or log10 CFU/mL. Qualitative culture-screening results were summarised descriptively and were not included in ANOVA. Results below the LOQ were treated as left-censored observations. When all five results in a category were below the LOQ, the category was reported as <10 CFU/g or <10 CFU/mL and no mean or standard deviation was calculated. Records with counts > 300 CFU/g were treated as right-censored observations. They were not assigned exact numerical values and were excluded from means, standard deviations and ANOVA. For Liqenasi and Bardhoke, the lower bound established that all five *Escherichia coli* results exceeded m = 100 CFU/g, whereas the number exceeding M = 1000 CFU/g remained indeterminate; any sampling-plan interpretation was therefore contextual and contingent on the criterion being applicable at the sampled stage. The traditional-cheese ANOVA included only Pogradeci, Vlora and Hasi, for which all individual Escherichia coli results were quantified. Individual results and the handling of censored observations are provided in Supplementary Table S6.

All statistical computations were performed in Python 3.13.5 using SciPy 1.17.0 and statsmodels 0.14.6. Exploratory one-way ANOVA was used to compare log10-transformed counts among the three sampled raw-milk goat-cheese origin groups with fully quantified values and among the three sampled raw cow milk farms. Because each group contained five independent experimental units, all inferential results were interpreted as exploratory. Residual normality was assessed with the Shapiro–Wilk test and homogeneity of variance with the median-centred Levene test, reported as the Brown–Forsythe test. Welch ANOVA and the Kruskal–Wallis test were used as robustness checks. Tukey’s HSD test was used for post hoc comparisons after a conventional ANOVA. Statistical significance was set at *p* < 0.05. Effect size was reported as eta squared and omega-squared. Ninety-five percent confidence intervals for eta squared were calculated by stratified percentile bootstrap with 100,000 resamples and random seed 20260623. Software versions, assumption-test outcomes, effect sizes, confidence intervals, post hoc results and robustness checks are provided in Supplementary Table S7.

## 3. Results and Discussion

### 3.1. Indicator Microorganisms

Among the sampled independent product lots, cow milk feta-type brined cheese and sausage had beta-glucuronidase-positive Escherichia coli counts below 10 CFU/g, while goat yoghurt and pistachio cake had Enterobacteriaceae counts below 10 CFU/g (Table 1). These findings apply only to the sampled lots. Mean Escherichia coli counts for the three fully quantified raw-milk goat-cheese origin groups were 3.25 ± 0.04 log CFU/g for Pogradeci, 3.17 ± 0.07 log CFU/g for Hasi and 3.08 ± 0.06 log CFU/g for Vlora. The exploratory one-way ANOVA identified differences among the three sampled origin groups (*F*(2,12) = 11.43, *p* = 0.00167; η^2^ = 0.656; ω^2^ = 0.582; bootstrap 95% CI for η^2^, 0.533–0.875). Tukey HSD separated Pogradeci from Vlora (*p* = 0.00121), whereas Pogradeci versus Hasi (*p* = 0.106) and Hasi versus Vlora (*p* = 0.0616) were not significant. Welch ANOVA (F(2,7.43) = 15.39, *p* = 0.00228) and the Kruskal–Wallis test (H = 8.88, *p* = 0.0118) supported differences among the sampled origin groups. For Liqenasi and Bardhoke, all five results in each group were >300 CFU/g. These right-censored observations were excluded from ANOVA. Pogradeci, Vlora and Hasi cheeses were made from raw milk, so the EU Escherichia coli process-hygiene criterion for cheeses made from heat-treated milk or whey was not applied to these groups. Liqenasi and Bardhoke were made from pasteurised milk; all five results in each group exceeded m = 100 CFU/g, so each five-sample set would be unsatisfactory if the criterion was applied at the sampled stage, while the number of results above M = 1000 CFU/g could not be determined. Because origin was not experimentally separated from producer-, process-, or lot-specific factors, no causal interpretation is warranted. All comparisons remain limited to the sampled products. This pattern is consistent with studies from other Balkan and Mediterranean artisanal cheese systems, where microbial quality is strongly affected by raw material quality, manual curd handling, ripening conditions and the degree of production standardisation [7,8,12,14].

The lower indicator counts in cow milk feta-type brined cheese are consistent with a more standardised product category, including controlled milk treatment, brining and refrigerated storage, but this interpretation remains explanatory rather than confirmatory because no structured audit of the production process was performed. Elevated Escherichia coli counts in traditional cheeses should be interpreted primarily as process hygiene signals, not as direct evidence of pathogen presence. Possible points for follow-up include contact surfaces, water quality, curd cutting and drainage, moulding, brining and temperature control before ripening. Studies on raw and thermally treated milk cheeses show that *Escherichia coli* levels are influenced by milk treatment, cheese type and handling intensity [23]. For the Albanian cheeses examined here, the results suggest that small changes in routine practice, especially improved cleaning verification and stricter control of handling and cooling, may substantially reduce indicator counts without undermining the products’ traditional character.

### 3.2. Pathogenic Bacteria

Unconfirmed presumptive *Salmonella* spp. culture-screening findings were obtained for all five frozen-chicken portions tested at 10 g and all five pork portions tested at 25 g (Table 1). The frozen-chicken findings cannot be interpreted against criteria that require the absence of 25 g. The pork findings were obtained from 25 g portions, but formal EU interpretation still depends on the exact product category and complete confirmatory identification. Possible contamination routes include slaughter, cutting, portioning, packaging, freezing or thawing and retail handling, but these remain hypotheses because process observations, environmental testing and complete cold-chain records were not available.

These unconfirmed presumptive *Salmonella* spp. culture-screening findings constitute food-safety screening signals rather than confirmed organism-level or epidemiological findings [24,25]. Selective plating and basic biochemical screening were performed, but complete confirmatory identification, serotyping and molecular characterisation were not undertaken. The results, therefore, do not support conclusions about *Salmonella* species, serotype, source attribution or antimicrobial resistance.

*Listeria monocytogenes* was not detected in 25 g in any of the five grilled chicken fillet samples. The product was a cooked, ready-to-eat food with a declared shelf life of 14 days. The exact shelf-life day on which the samples were tested was not recorded. The result, therefore, supports absence only in the tested portions and does not demonstrate absence throughout shelf life, effective control of post-process contamination or inability of the product to support growth. Commission Regulation (EU) 2024/2895, applicable from 1 July 2026, is discussed only as relevant regulatory context [6].

Coagulase-positive staphylococci were below the LOQ of 10 CFU/g in all five samples from each tested cheese group. These findings provide process-hygiene information only. They do not exclude a staphylococcal enterotoxin hazard because enterotoxins were not analysed and because the applicability of the criterion depends on the specific cheese category and manufacturing stage [4,9,26].

### 3.3. Total Mesophilic Aerobic Counts (TMAC)

For traditional cheeses, all five results in each of the five total aerobic colony-count categories were >300 CFU/g. These observations were right-censored and do not represent exact concentrations above 300 CFU/g. Means and standard deviations were therefore not calculated, and the results were used only to show that all 25 independent product-lot samples exceeded the reporting threshold.

Raw milk gave a different message. Total aerobic colony counts ranged from 4.52 to 6.52 log CFU/mL and differed among the three sampled farm groups. The exploratory one-way ANOVA gave F(2,12) = 5928.00, *p* = 1.07 × 10^−18^, with η^2^ = 0.99899, ω^2^ = 0.99874 and a bootstrap 95% CI for η^2^ of 0.99875–0.99963. Tukey HSD found all pairwise contrasts significant at *p* < 0.001, with Farm 1 assigned group a, Farm 3 group b and Farm 2 group c. Welch ANOVA gave F(2,7.60) = 6059.39, *p* = 6.64 × 10^−13^, and the Kruskal–Wallis test gave H = 12.50, *p* = 0.00193. The mean values for Farm 1 and Farm 3 were above the 5.0 log CFU/mL benchmark, equivalent to 100,000 CFU/mL, while the mean value for Farm 2 was below this benchmark. These comparisons are screening evidence only. Formal assessment under Regulation (EC) No 853/2004 requires a rolling geometric average over two months with at least two samples per month [5].

Since no structured farm audit was conducted, the study cannot identify the precise cause of the differences among the sampled farm groups. The results suggest that follow-up work should combine microbiological testing with assessment of cooling capacity, water quality, udder hygiene and equipment sanitation.

### 3.4. EU-Aligned Interpretation and Limitations

The results were considered in relation to Commission Regulation (EC) No 2073/2005 [4] and Regulation (EC) No 853/2004 [5] only where the product category, sampling stage, test portion and result format permitted such interpretation. The EU criteria were not used to convert incomplete laboratory records into formal compliance or non-compliance decisions. Detailed qualifications are provided in Supplementary Table S2.

The study has several limitations. The sampling was targeted, and 145 physical samples from independent product lots or independent bulk-milk lots, obtained in separate collection events, cannot be used to estimate the national prevalence. Each of the 29 analytical sampling categories contained five independent experimental units; repeated product or origin names represented separately collected sets rather than repeated analyses of the same material. Results reported as >300 CFU/g were right-censored, and exact counts above this value were not quantified. The unconfirmed presumptive *Salmonella* spp. culture-screening findings were based on selective culture and basic biochemical screening without complete confirmatory identification, serotyping or molecular characterisation. *Listeria monocytogenes* was tested only in grilled chicken fillet, and coagulase-positive staphylococci were not tested for enterotoxins. No structured audit was performed at farms, processing sites or retail points.

The EU *Escherichia coli* process-hygiene criterion with *n* = 5, c = 2, m = 100 CFU/g, and M = 1000 CFU/g applies to cheeses made from milk or whey that have undergone heat treatment and at the manufacturing stage when the *Escherichia coli* count is expected to be highest [4]. Pogradeci, Vlora and Hasi cheeses were made from raw milk and were therefore not classified against this criterion. Liqenasi and Bardhoke cheeses were made from pasteurised milk and were sampled at the production site after manufacture. All five results in each group were >300 CFU/g, and therefore above the m = 100 CFU/g threshold. Because c = 2, each five-sample set would be unsatisfactory if the criterion were applied at the sampling stage, while the number of results above M = 1000 CFU/g could not be determined from the right-censored values. Cow milk feta-type brined cheese was made from pasteurised milk and yielded <10 CFU/g, but the exact point at which the maximum expected Escherichia coli count would be reached was not established. These criterion-based interpretations are therefore contextual rather than formal enforcement classifications. For raw milk, all five Farm 1 and Farm 3 results were above 100,000 CFU/mL, whereas all five Farm 2 results were below this value. Formal assessment still requires the rolling geometric-average framework specified in Regulation (EC) No 853/2004 [5].

The unconfirmed presumptive *Salmonella* spp. culture-screening findings in raw poultry and pork should be treated as food-safety concerns that require repeat sampling and definitive confirmation [4]. The broader technological interpretation remains cautious. The data identify follow-up priorities rather than proven contamination routes, including slaughter and portioning hygiene for meat, cold-chain management for raw milk, and hygienic handling during artisanal cheese manufacture.

Taken together, the data make the study relevant as a targeted case study of selected small-scale Albanian food chains under EU-aligned safety expectations. The pattern observed here may be relevant to comparable small-scale food systems, but this study does not demonstrate that pattern outside the sampled Albanian product lots. The practical contribution is the identification of follow-up priorities to which limited resources can be directed first: raw milk cooling and equipment hygiene; verification of water and contact-surface sanitation in cheesemaking; hygienic curd handling; and prevention of cross-contamination during meat cutting and retail display.

## 4. Conclusions

This study provides a targeted microbiological assessment of 145 physical samples representing independent product lots or independent bulk-milk lots obtained in separate collection events, organised into 29 organism-by-matrix analytical categories. Cow milk feta-type brined cheese, goat yoghurt, cooked ready-to-eat sausage and pistachio cake showed low *Escherichia coli* or Enterobacteriaceae counts in the sampled lots. Three raw-milk traditional cheese groups had quantified *Escherichia coli* counts from 3.08 to 3.25 log CFU/g. In Liqenasi and Bardhoke, all five *Escherichia coli* results in each category were >300 CFU/g and were right-censored, so exact means could not be calculated. Raw milk displayed differences among the sampled farms in total aerobic colony counts.

The most important food-safety signal was the occurrence of unconfirmed presumptive Salmonella spp. culture-screening findings in frozen chicken and pork. Because the findings were based on selective culture and basic biochemical screening without complete confirmatory identification or serotyping, and because frozen chicken was tested in 10 g, they require repeat sampling and full confirmation. *Listeria monocytogenes* was not detected in the grilled chicken portions tested, but this result should not be extended beyond the tested samples or interpreted as shelf-life assurance.

For the dairy chain, the findings suggest follow-up targets rather than confirmed causes. These include raw milk cooling, milking equipment sanitation, hygienic curd handling, and control of brining, contact surfaces, and storage temperature. Future work should use larger, fully documented sampling frames, use dilution schemes that permit quantification of results above 300 CFU/g, report individual results, and perform complete confirmatory pathogen identification and structured process audits.

## Figures and Tables

**Table 1:** Product-level summary of the main microbiological findings. Each five-unit set comprised independent product lots or, for raw milk, independent bulk-milk lots obtained in separate collection events. Detailed sampling, methods, individual values and regulatory qualifications are provided in Supplementary Tables S1–S7.

Matrix or Product Category	Endpoint	Reported Finding in Tested Samples	EU-Aligned Interpretation	Main Limitation
Cow milk feta-type brined cheese	Beta-glucuronidase-positive *Escherichia coli*	<10 CFU/g in all five samples	Low process-hygiene indicator count in the tested samples	Pasteurised-milk product sampled after manufacture; the exact maximum-count stage required by the criterion was not established
Goat yoghurt	Enterobacteriaceae	<10 CFU/g in all five samples	Descriptive process-hygiene information	No product-specific EU criterion was applied
Cooked ready-to-eat sausage	Beta-glucuronidase-positive *Escherichia coli*	<10 CFU/g in all five samples	Low indicator count in the tested samples	No product-specific EU criterion was applied
Pistachio cake	Enterobacteriaceae	<10 CFU/g in all five samples	Descriptive hygiene information	No formal EU classification was made
Traditional cheeses, five sampled origin groups	*Escherichia coli*	Pogradeci: 1600–2000 CFU/g; Vlora: 1000–00 CFU/g; Hasi: 1200–1800 CFU/g; Liqenasi and Bardhoke: >300 CFU/g in all five independent product lots per group	Raw-milk groups were interpreted descriptively. For Liqenasi and Bardhoke, all five results exceeded m = 100 CFU/g; each set would be unsatisfactory if the criterion was applied at the sampled stage	Exact counts above 300 CFU/g were not quantified; the number above M = 1000 CFU/g was indeterminate and criterion applicability remained contextual
Traditional goat cheeses, four sampled origin groups	Coliforms	Liqenasi, Pogradeci and Vlora: <10 CFU/g in all five product lots per group; Hasi: >300 CFU/g in all five product lots	Descriptive hygiene information only	The Hasi results were right-censored; exact counts above 300 CFU/g were not quantified
Frozen chicken fillet	Unconfirmed presumptive *Salmonella* spp. culture-screening finding	Finding in 10 g for all five independent product lots	Food-safety screening signal	A 10 g test portion and incomplete confirmation prevent formal absence-in-25 g or serotype-specific interpretation
Pork fillet	Unconfirmed presumptive *Salmonella* spp. culture-screening finding	Finding in 25 g for all five independent product lots	Food-safety screening signal	Formal interpretation requires the exact product category and complete confirmatory identification
Grilled chicken fillet	*Listeria monocytogenes*	Not detected in 25 g in 5/5 samples	Absence in the tested portions only	Ready-to-eat product with 14-day shelf life
Traditional cheeses	Coagulase-positive staphylococci	<10 CFU/g in all tested samples	Process-hygiene information only	Criterion depends on cheese category and manufacturing stage; enterotoxins were not analysed
Traditional cheeses	Total aerobic colony count at 30 °C	All five results in each of five independently sampled cheese categories were >300 CFU/g	Right-censored results; no numerical mean or standard deviation was calculated	Exact counts above 300 CFU/g were not quantified
Raw cow milk, Farm 1	Total aerobic colony count at 30 °C	6.52 ± 0.02 log CFU/mL	Mean above the 100,000 CFU/mL benchmark	Formal classification requires the prescribed rolling geometric average
Raw cow milk, Farm 2	Total aerobic colony count at 30 °C	4.52 ± 0.03 log CFU/mL	Mean below the 100,000 CFU/mL benchmark	Formal classification requires the prescribed rolling geometric average
Raw cow milk, Farm 3	Total aerobic colony count at 30 °C	5.64 ± 0.03 log CFU/mL	Mean above the 100,000 CFU/mL benchmark	Formal classification requires the prescribed rolling geometric average

## Data Availability

The original contributions presented in this study are included in the article/Supplementary Material. Further inquiries can be directed to the corresponding authors.

## Supplementary Materials

The following supporting information can be downloaded at https://www.mdpi.com/article/10.3390/foods15132335/s1: Table S1. Expanded ISO methods and reporting details; Table S2. EU microbiological criteria or benchmarks used for interpretation; Table S3. Complete sampling frame for the 29 analytical sampling categories. Each category comprised five experimental units from five independent product lots or, for raw milk, five independent bulk-milk lots, each obtained in a separate collection event, yielding 145 physical samples in total. Repeated product names in different rows represent separate five-unit sets analysed for different endpoints. Lot codes and producer identities are withheld to protect commercial confidentiality; Table S4. Product-characterisation data needed for technological and EU-criterion interpretation; Table S5. Organism-by-matrix testing plan and rationale; Table S6. Individual sample results and censored values. All rows contain results from five independent product lots or, for raw milk, five independent bulk-milk lots, each obtained in a separate collection event. Values <10 CFU/g are left-censored at the LOQ. For values reported as >300 CFU/g, 1.0 mL of the 1:10 initial suspension represented 0.1 g, and >30 colonies therefore corresponded to >300 CFU/g. Exact counts above this reporting category were not retained; all such values remain right-censored and have no numerical mean or standard deviation; Table S7. Statistical analysis details and robustness checks. Conventional ANOVA was restricted to exact numerical results; datasets reported only as >300 CFU/g were treated as right-censored and were not entered into these analyses.
