# Supplementary material for: Microbiological Safety of Selected Albanian Foods in Relation to EU Criteria: Evidence from Artisanal Dairy and Meat Supply Chains
Source: Foods. 2026 Jul 1;15(13):2335. doi: 10.3390/foods15132335 (PMC13361519; doi:10.3390/foods15132335)
Supplement: Supplementary file 1 [file foods-15-02335-s001.zip › foods-4377313-supplementary.pdf]

Microbiological Safety of Selected Albanian Foods in Relation to EU Criteria: Evidence from Artisanal Dairy and Meat Supply Chains

Supplementary Tables

Table S1. Expanded ISO methods and reporting details.

Target / parameter	ISO method	Test portion and preparation	Media and incubation	Result type and LOQ	Confirmation and documented quality-control information
Beta-glucuronidase-positive *Escherichia coli*	ISO 16649-2:2001	10 g or 10 mL sample plus 90 mL diluent. Diluent type: Peptone Water according to ISO 6887. Homogenisation time: 2 minutes using a stomacher. Decimal dilutions prepared as required.	TBX agar; incubation at 44 °C for 18 to 24 h.	Enumeration, CFU/g or CFU/mL. LOQ 10 CFU/g or mL. For results reported as >300 CFU/g, 1.0 mL of the 1:10 initial suspension represented 0.1 g; >30 colonies therefore corresponded to >300 CFU/g.	Blue-green colonies on TBX agar were enumerated as beta-glucuronidase-positive Escherichia coli. No additional species-level confirmation was performed. Exact colony counts above 30 in the plated aliquot were not recorded.
Enterobacteriaceae	ISO 21528-2:2017	25 g test portion is homogenized with 225 mL volumes of a suitable diluent (Buffered Peptone Water according to the ISO 6887 series) using a peristaltic blender for 2 minutes. Decimal dilutions prepared as required.	VRBG pour plates with an overlay; incubation at 37 °C ± 1 °C for 24 h ± 2 h.	Enumeration, CFU/g or CFU/mL. LOQ 10 CFU/g or mL.	Typical colonies were subjected to basic oxidase and glucose-fermentation checks. The complete colony-selection record and detailed control-strain documentation were not retained.

Target / parameter	ISO method	Test portion and preparation	Media and incubation	Result type and LOQ	Confirmation and documented quality-control information
Unconfirmed presumptive *Salmonella* spp. culture-screening finding	ISO 6579-1:2017-based culture screening	Frozen chicken fillet tested in 10 g; pork fillet tested in 25 g. Pre-enrichment in Buffered Peptone Water (BPW) using a 1:10 ratio (10 g sample + 90 mL BPW; 25 g sample + 225 mL BPW).	1. Non-selective pre-enrichment: BPW incubated at 34–38 °C for 18 ± 2 h.<br>2. Selective enrichment: 0.1 mL of BPW culture into 10 mL of Rappaport-Vassiliadis Soya (RVS) broth (incubated at 41.5 °C for 24 ± 3 h) and 1 mL of BPW culture into 10 mL of Muller-Kauffmann Tetrathionate-Novobiocin (MKTTn) broth (incubated at 37 °C for 24 ± 3 h).<br>3. Selective plating: Inoculation onto Xylose Lysine Deoxycholate (XLD) agar (incubated at 37 °C for 24 ± 3 h) and a second complementary selective agar (BGA) incubated according to manufacturer instructions.	Unconfirmed presumptive culture-screening finding or no such finding in the specified test portion. LOQ not applicable.	The finding was based on typical colonies on selective media and the basic biochemical screening retained in the laboratory records. The complete biochemical panel was not recorded. Serological agglutination, species-level identification, serotyping and molecular characterisation were not performed.
Listeria monocytogenes	ISO 11290-1:2017	Grilled chicken fillet tested in 25 g. Test portion (25 g) is diluted in 225 mL of Half-Fraser broth (1:10 dilution) and homogenized (2 min.).	Primary enrichment: Half-Fraser broth, incubated at 30 °C ± 1 °C for 25 h ± 1 h.<br>Secondary enrichment: 0.1 mL into 10 mL Fraser broth at 37 °C ± 1 °C for 24 h ± 2 h. Selective plating from both enrichments on ALOA and Oxford agar at 37 °C ± 1 °C for 24 h ± 2 h, extended to 48 h ± 2 h.	Detected or not detected in 25 g. LOQ not applicable.	No presumptive colonies requiring confirmation were observed.

Target / parameter	ISO method	Test portion and preparation	Media and incubation	Result type and LOQ	Confirmation and documented quality-control information
Coagulase-positive staphylococci	ISO 6888-2:2021/Amd 1:2023	25 g test portion mixed at a 1:10 ratio with a suitable diluent, such as buffered peptone water, and homogenised in a peristaltic blender for 2 min. Decimal dilutions were prepared as required.	Rabbit plasma fibrinogen agar medium according to ISO method. Aerobic incubation at 34 °C to 38 °C for 24 h ± 2 h (and if necessary, for a further 24 h ± 2 h)	Enumeration, CFU/g. LOQ 10 CFU/g; results below the LOQ reported as <10 CFU/g.	Results were reported as coagulase-positive staphylococci. Staphylococcal enterotoxins were not analysed.
Total aerobic colony count at 30 °C	ISO 4833:2003	10 g or 10 mL of the sample. Diluent: Buffered peptone water or other appropriate diluent as specified in the relevant part of ISO 6887. Dilution scheme: 1 mL of the initial suspension (and subsequent decimal dilutions) is inoculated into empty Petri dishes in duplicate.	Plate count agar, 30 °C according to ISO method. Incubation time: 72 h ± 3 h	Enumeration, CFU/g or CFU/mL. LOQ 10 CFU/g or mL. One millilitre of the 1:10 initial suspension represented 0.1 g; >30 colonies corresponded to >300 CFU/g.	Results were expressed as CFU/g or CFU/mL. Exact colony counts above 30 in the plated aliquot were not recorded; these results were treated as right-censored.
Coliforms	ISO 4832:2006	Enumeration performed via decimal dilutions and pour-plate technique.	Crystal violet neutral red bile lactose (VRBL) agar. Incubation at 37 °C for 24 h	Enumeration, CFU/g. LOQ 10 CFU/g. One millilitre of the 1:10 initial suspension represented 0.1 g; >30 colonies corresponded to >300 CFU/g.	Typical colonies were enumerated. Exact colony counts above 30 in the plated aliquot were not recorded; values >300 CFU/g were treated as right-censored.

Table S2. EU microbiological criteria or benchmarks used for interpretation

Product / matrix	Microorganism or parameter	EU source	Criterion / benchmark	Use in this study
Cheeses made from milk or whey that has undergone heat treatment	*Escherichia coli*	Commission Regulation (EC) No 2073/2005	n = 5, c = 2, m = 100 CFU/g, M = 1000 CFU/g. The criterion applies at the manufacturing stage when the *Escherichia coli* count is expected to be highest.	Not applied to raw-milk Pogradeci, Vlora or Hasi goat cheeses. For pasteurised-milk Liqenasi goat cheese and Bardhoke sheep cheese, all five results in each category were >300 CFU/g and therefore above m = 100 CFU/g. Because c = 2, each five-sample set would be unsatisfactory if the criterion were applicable at the sampled stage. The number of results above M = 1000 CFU/g could not be determined. Interpretation remains contextual because the stage of maximum expected Escherichia coli count was not established. For cow-milk feta-type brined cheese, all five results were <10 CFU/g.
Raw cows milk intended for further processing	Total bacterial count at 30 °C	Regulation (EC) No 853/2004, Annex III, Section IX	≤100,000 CFU/mL as rolling geometric average under regulatory sampling rules	Used only as benchmark because this study did not apply the full rolling sampling framework.
Poultry meat preparations, mechanically separated meat or fresh poultry meat where applicable	Unconfirmed presumptive *Salmonella* spp. culture-screening finding	Commission Regulation (EC) No 2073/2005	Absence in specified test portion; selected poultry criteria require serotype information	Unconfirmed presumptive culture-screening findings were treated as food-safety screening signals. Formal interpretation requires the exact product category, a 25 g test portion where applicable and serotyping.
Pork, minced meat, meat preparations or meat products where applicable	Unconfirmed presumptive *Salmonella* spp. culture-screening finding	Commission Regulation (EC) No 2073/2005	Absence in specified test portion for relevant product categories	The pork findings were treated as screening signals. Formal interpretation depends on the product category and complete confirmation.
Ready-to-eat foods such as grilled chicken where applicable	*Listeria monocytogenes*	Commission Regulation (EC) No 2073/2005; Commission Regulation (EU) 2024/2895	Not detected in 25 g or ≤100 CFU/g depending on product status, shelf-life evidence and date of application. Amendment applies from July 2026 for RTE foods able to support growth.	Negative result interpreted only for the five tested 25 g portions. The product had a declared 14-day shelf life, but sampling day, pH, water activity and growth potential were not determined.

Selected dairy products where applicable	Coagulase-positive staphylococci	Commission Regulation (EC) No 2073/2005	m/M values depend on specific dairy category	Used as process hygiene information only. Enterotoxins were not analysed.

Table S3. Complete sampling frame for the 29 analytical sampling categories. Each category comprised five experimental units from five independent product lots or, for raw milk, five independent bulk-milk lots, each obtained in a separate collection event, yielding 145 physical samples in total. Repeated product names in different rows represent separate five-unit sets analysed for different endpoints. Lot codes and producer identities are withheld to protect commercial confidentiality.

No.	Analytical sampling category	Microorganism / parameter	Origin or source	Collection site type	Collection period	Experimental unit and independence	Storage and ready-to-eat status
1	Goat cheese: Pogradeci	β-glucuronidase-positive *E. coli*	Pogradeci, Albania	Production site, after manufacture	January 2025	Five physical samples, each from a different production lot. Lot codes and producer identity are withheld. No physical sample was reused in another analytical category.	Storage temperature 4-8 °C Ready-to-eat product
2	Goat cheese: Vlora	β-glucuronidase-positive *E. coli*	Vlora, Albania	Production site, after manufacture	January 2025	Five physical samples, each from a different production lot. Lot codes and producer identity are withheld. No physical sample was reused in another analytical category.	Storage temperature 4-8 °C Ready-to-eat product
3	Goat cheese: Hasi	β-glucuronidase-positive *E. coli*	Hasi, Albania	Production site, after manufacture	February 2025	Five physical samples, each from a different production lot. Lot codes and producer identity are withheld. No physical sample was reused in another analytical category.	Storage temperature 4-8 °C Ready-to-eat product
4	Goat cheese: Liqenasi	β-glucuronidase-positive *E. coli*	Liqenasi, Albania	Production site, after manufacture	February 2025	Five physical samples, each from a different production lot. Lot codes and producer identity are withheld. No physical sample was reused in another analytical category.	Storage temperature 4-8 °C Ready-to-eat product
5	Sheep cheese: Bardhoke	β-glucuronidase-positive *E. coli*	Bardhoke, Albania	Production site, after manufacture	March 2025	Five physical samples, each from a different production lot. Lot codes and producer identity are withheld. No physical sample was reused in another analytical category.	Storage temperature 4-8 °C Ready-to-eat product

No.	Analytical sampling category	Microorganism / parameter	Origin or source	Collection site type	Collection period	Experimental unit and independence	Storage and ready-to-eat status
6	Cow milk feta-type brined cheese	Beta-glucuronidase-positive *Escherichia coli*	Tirana, Albania	Production site, after manufacture	March 2025	Five physical samples, each from a different production lot. Lot codes and producer identity are withheld. No physical sample was reused in another analytical category.	Storage temperature 4-8 °C Ready-to-eat product
7	Goat yoghurt	Enterobacteriaceae	Vlora, Albania	Retail	April 2025	Five physical samples, each from a different production lot. Lot codes and producer identity are withheld. No physical sample was reused in another analytical category.	Storage temperature 4-8 °C Ready-to-eat product
8	Sausage	Beta-glucuronidase-positive *Escherichia coli*	Korca, Albania	Market	April 2025	Five physical samples, each from a different production lot. Lot codes and producer identity are withheld. No physical sample was reused in another analytical category.	Cooked ready-to-eat product; storage 4–8 °C
9	Pistachio cake	Enterobacteriaceae	Tirana, Albania	Market	April 2025	Five physical samples, each from a different production lot. Lot codes and producer identity are withheld. No physical sample was reused in another analytical category.	Storage temperature 4-8 °C Ready-to-eat product
10	Raw cow milk: Farm 1	Total aerobic colony count at 30 °C	Anonymised Farm 1, Tirana, Albania	Farm / primary production	May 2025	Five physical samples from five independent bulk-milk lots, each obtained in a separate collection event. Farm identity and internal sample codes are withheld. No physical sample was reused in another analytical category.	Raw milk, intended for further processing. Storage temperature 4-8 °C

No.	Analytical sampling category	Microorganism / parameter	Origin or source	Collection site type	Collection period	Experimental unit and independence	Storage and ready-to-eat status
11	Raw cow milk: Farm 2	Total aerobic colony count at 30 °C	Anonymised Farm 2, Kruja, Albania	Farm / primary production	May 2025	Five physical samples from five independent bulk-milk lots, each obtained in a separate collection event. Farm identity and internal sample codes are withheld. No physical sample was reused in another analytical category.	Raw milk, intended for further processing. Storage temperature 4-8 °C
12	Raw cow milk: Farm 3	Total aerobic colony count at 30 °C	Anonymised Farm 3, Kavaja, Albania	Farm / primary production	May 2025	Five physical samples from five independent bulk-milk lots, each obtained in a separate collection event. Farm identity and internal sample codes are withheld. No physical sample was reused in another analytical category.	Raw milk, intended for further processing. Storage temperature 4-8 °C
13	Frozen chicken fillet	Unconfirmed presumptive *Salmonella* spp. culture-screening finding	Fier, Tirana	Market	June 2025	Five physical samples, each from a different production lot. Lot codes and producer identity are withheld. No physical sample was reused in another analytical category.	Frozen raw product, not ready-to-eat; storage and transport at −18 °C
14	Pork fillet	Unconfirmed presumptive *Salmonella* spp. culture-screening finding	Lezhe, Albania	Market	June 2025	Five physical samples, each from a different production lot. Lot codes and producer identity are withheld. No physical sample was reused in another analytical category.	Raw chilled product, not ready-to-eat
15	Grilled chicken fillet	*Listeria monocytogenes*	Tirana, Albania	Market	September 2025	Five physical samples, each from a different production lot. Lot codes and producer identity are withheld. No physical sample was reused in another analytical category.	Ready-to-eat grilled product; declared shelf life 14 days; storage 4–8 °C

No.	Analytical sampling category	Microorganism / parameter	Origin or source	Collection site type	Collection period	Experimental unit and independence	Storage and ready-to-eat status
16	Goat cheese: Liqenasi	Coagulase-positive staphylococci	Liqenasi, Albania	Production site, after manufacture	2025–2026; exact collection date not retained	Five physical samples, each from a different production lot. Lot codes and producer identity are withheld. No physical sample was reused in another analytical category.	Storage temperature 4-8 °C Ready-to-eat product
17	Goat cheese: Liqenasi	Coliforms	Liqenasi, Albania	Production site, after manufacture	2025–2026; exact collection date not retained	Five physical samples, each from a different production lot. Lot codes and producer identity are withheld. No physical sample was reused in another analytical category.	Storage temperature 4-8 °C Ready-to-eat product
18	Goat cheese: Pogradeci	Coliforms	Pogradeci, Albania	Production site, after manufacture	2025–2026; exact collection date not retained	Five physical samples, each from a different production lot. Lot codes and producer identity are withheld. No physical sample was reused in another analytical category.	Storage temperature 4-8 °C Ready-to-eat product
19	Goat cheese: Vlora	Coliforms	Vlora, Albania	Production site, after manufacture	2025–2026; exact collection date not retained	Five physical samples, each from a different production lot. Lot codes and producer identity are withheld. No physical sample was reused in another analytical category.	Storage temperature 4-8 °C Ready-to-eat product
20	Goat cheese: Hasi	Coliforms	Hasi, Albania	Production site, after manufacture	2025–2026; exact collection date not retained	Five physical samples, each from a different production lot. Lot codes and producer identity are withheld. No physical sample was reused in another analytical category.	Storage temperature 4-8 °C Ready-to-eat product
21	Goat cheese: Liqenasi	Total aerobic colony count at 30 °C	Liqenasi, Albania	Production site, after manufacture	2025–2026; exact collection date not retained	Five physical samples, each from a different production lot. Lot codes and producer identity are withheld. No physical sample was reused in another analytical category.	Storage temperature 4-8 °C Ready-to-eat product

No.	Analytical sampling category	Microorganism / parameter	Origin or source	Collection site type	Collection period	Experimental unit and independence	Storage and ready-to-eat status
22	Goat cheese: Pogradeci	Total aerobic colony count at 30 °C	Pogradeci, Albania	Production site, after manufacture	2025–2026; exact collection date not retained	Five physical samples, each from a different production lot. Lot codes and producer identity are withheld. No physical sample was reused in another analytical category.	Storage temperature 4-8 °C Ready-to-eat product
23	Goat cheese: Vlora	Total aerobic colony count at 30 °C	Vlora, Albania	Production site, after manufacture	2025–2026; exact collection date not retained	Five physical samples, each from a different production lot. Lot codes and producer identity are withheld. No physical sample was reused in another analytical category.	Storage temperature 4-8 °C Ready-to-eat product
24	Goat cheese: Hasi	Total aerobic colony count at 30 °C	Hasi, Albania	Production site, after manufacture	2025–2026; exact collection date not retained	Five physical samples, each from a different production lot. Lot codes and producer identity are withheld. No physical sample was reused in another analytical category.	Storage temperature 4-8 °C Ready-to-eat product
25	Sheep cheese: Bardhoke	Total aerobic colony count at 30 °C	Bardhoke, Albania	Production site, after manufacture	2025–2026; exact collection date not retained	Five physical samples, each from a different production lot. Lot codes and producer identity are withheld. No physical sample was reused in another analytical category.	Storage temperature 4-8 °C Ready-to-eat product
26	Goat cheese: Pogradeci	Coagulase-positive staphylococci	Pogradeci, Albania	Production site, after manufacture	2025–2026; exact collection date not retained	Five physical samples, each from a different production lot. Lot codes and producer identity are withheld. No physical sample was reused in another analytical category.	Storage temperature 4-8 °C Ready-to-eat product
27	Goat cheese: Vlora	Coagulase-positive staphylococci	Vlora, Albania	Production site, after manufacture	2025–2026; exact collection date not retained	Five physical samples, each from a different production lot. Lot codes and producer identity are withheld. No physical sample was reused in another analytical category.	Storage temperature 4-8 °C Ready-to-eat product

No.	Analytical sampling category	Microorganism / parameter	Origin or source	Collection site type	Collection period	Experimental unit and independence	Storage and ready-to-eat status
28	Goat cheese: Hasi	Coagulase-positive staphylococci	Hasi, Albania	Production site, after manufacture	2025–2026; exact collection date not retained	Five physical samples, each from a different production lot. Lot codes and producer identity are withheld. No physical sample was reused in another analytical category.	Storage temperature 4-8 °C Ready-to-eat product
29	Sheep cheese: Bardhoke	Coagulase-positive staphylococci	Bardhoke, Albania	Production site, after manufacture	2025–2026; exact collection date not retained	Five physical samples, each from a different production lot. Lot codes and producer identity are withheld. No physical sample was reused in another analytical category.	Storage temperature 4-8 °C Ready-to-eat product

Table S4. Product-characterisation data needed for technological and EU-criterion interpretation.

Product / matrix	Milk or thermal status	Ripening / brine / salt conditions	pH	Water activity	Packaging	Storage temperature and shelf-life stage	Intended use
Goat cheese: Pogradeci	raw milk	ripening time in brine 2 month in 12% salt	Not measured in this study	Not measured in this study	In a plastic box with brine	Storage temperature 4-8 °C; Shelf life 3 month	Ready-to-eat product
Goat cheese: Vlora	raw milk	ripening time in brine 2 month in 12% salt	Not measured in this study	Not measured in this study	In a plastic box with brine	Storage temperature 4-8 °C; Shelf life 3 month	Ready-to-eat product
Goat cheese: Hasi	raw milk	ripening time in brine 2 month in 12% salt	Not measured in this study	Not measured in this study	In a plastic box with brine	Storage temperature 4-8 °C; Shelf life 3 month	Ready-to-eat product
Goat cheese: Liqenasi	pasteurized milk	ripening time in brine 2 month in 12% salt	Not measured in this study	Not measured in this study	In a plastic box with brine	Storage temperature 4-8 °C; Shelf life 3 month	Ready-to-eat product
Sheep cheese: Bardhoke	pasteurized milk	ripening time in brine 2 month in 12% salt	Not measured in this study	Not measured in this study	In a plastic box with brine	Storage temperature 4-8 °C; Shelf life 3 month	Ready-to-eat product
Cow milk feta-type brined cheese	pasteurized milk	Brined cheese, ripening time in brine 2 month in 12% salt	Not measured in this study	Not measured in this study	In a plastic box with brine	Storage temperature 4-8 °C; Shelf life 3 month	Ready-to-eat product
Goat yoghurt	pasteurized milk	Fermented product; fermentation with backs loop. The inoculum composition and fermentation end point were not recorded.	Not measured in this study	Not measured in this study	In a plastic box	Storage temperature 4-8 °C; Shelf life 7 days	Ready-to-eat

Product / matrix	Milk or thermal status	Ripening / brine / salt conditions	pH	Water activity	Packaging	Storage temperature and shelf-life stage	Intended use
Frozen chicken fillet	Raw frozen meat	Not applicable	Not measured in this study	Not measured in this study	In plastic bag	Storage and transport at −18 °C; declared shelf life 3 months	Cook before consumption
Pork fillet	Raw meat	Not applicable	Not measured in this study	Not measured in this study	In plastic bag	Storage temperature 4-8 °C; Shelf life 14 days	Cook before consumption
Sausage	Cooked product	2.5% salt; exact heat-treatment parameters not retained	Not measured in this study	Not measured in this study	In plastic bag	Storage temperature 4-8 °C; Shelf life 14 days	Ready-to-eat
Grilled chicken fillet	Cooked meat product	Thermally treated	Not measured in this study	Not measured in this study	In plastic bag	Storage 4–8 °C; declared shelf life 14 days; exact sampling day within shelf life not retained	Ready-to-eat
Pistachio cake	Baked confectionery	Not applicable	Not measured in this study	Not measured in this study	In a cardboard box	Storage temperature 4-8 °C; Shelf life 3 days	Ready-to-eat
Raw cow milk: Farm 1	Raw milk	Not applicable	Not measured in this study	Not measured in this study	Bulk	Storage temperature 4-8 °C for 1 day	Further processing
Raw cow milk: Farm 2	Raw milk	Not applicable	Not measured in this study	Not measured in this study	Bulk	Storage temperature 4-8 °C for 1 day	Further processing
Raw cow milk: Farm 3	Raw milk	Not applicable	Not measured in this study	Not measured in this study	Bulk	Storage temperature 4-8 °C for 1 day	Further processing

Table S5. Organism-by-matrix testing plan and rationale.

Matrix / product group	Food safety endpoint tested	Process hygiene endpoint tested	Endpoint not tested or limited	Rationale / interpretation
Traditional cheeses	Not tested. Neither *Salmonella* spp. nor *Listeria monocytogenes* was analysed.	*Escherichia coli*, coagulase-positive staphylococci, coliforms where available, total aerobic colony count at 30 °C	*Salmonella* spp. and *Listeria monocytogenes* were not tested; staphylococcal enterotoxins were not analysed.	Focus was process hygiene and origin comparison. Missing pathogen testing limits food safety interpretation.
Cow milk feta-type brined cheese	Not tested.	Beta-glucuronidase-positive *Escherichia coli*	*Salmonella* spp., *Listeria monocytogenes*, coagulase-positive staphylococci and staphylococcal enterotoxins were not tested.	Used as more standardised dairy comparator.
Goat yoghurt	Not tested.	Enterobacteriaceae	*Salmonella* spp., *Listeria monocytogenes*, coagulase-positive staphylococci and staphylococcal enterotoxins were not tested.	Descriptive hygiene screening.
Raw cow milk farms	Not a ready-to-eat product in this study	Total aerobic colony count at 30 °C	Pathogens not reported in submitted dataset.	Farm-level screening against raw milk benchmark, not formal rolling compliance assessment.
Frozen chicken fillet	Unconfirmed presumptive *Salmonella* spp. culture-screening finding in 10 g	Not reported in submitted dataset	*Listeria monocytogenes* not reported. Indicator organisms not reported.	Raw-meat food-safety screening. The 10 g portion and incomplete confirmation limit EU absence-in-25 g interpretation.
Pork fillet	Unconfirmed presumptive *Salmonella* spp. culture-screening finding in 25 g	Not reported in submitted dataset	*Listeria monocytogenes* not reported. Indicator organisms not reported.	Raw-meat food-safety screening. Formal interpretation depends on product category and complete confirmation.

Matrix / product group	Food safety endpoint tested	Process hygiene endpoint tested	Endpoint not tested or limited	Rationale / interpretation
Grilled chicken fillet	*Listeria monocytogenes* in 25 g	Not reported in submitted dataset	*Salmonella spp.* not reported.	Ready-to-eat product screening limited to tested portions. Growth potential and shelf-life evidence required for full EU interpretation.
Sausage	Not tested.	Beta-glucuronidase-positive *Escherichia coli*	*Salmonella* spp. and *Listeria monocytogenes* were not tested; other process-hygiene indicators were not reported.	Cooked ready-to-eat sausage. The *Escherichia coli* result is descriptive process-hygiene information only.
Pistachio cake	Not tested.	Enterobacteriaceae	*Salmonella* spp., *Listeria monocytogenes* and coagulase-positive staphylococci were not tested.	Descriptive hygiene screening for ready-to-eat confectionery.

Table S6. Individual sample results and censored values. All rows contain results from five independent product lots or, for raw milk, five independent bulk-milk lots, each obtained in a separate collection event. Values <10 CFU/g are left-censored at the LOQ. For values reported as >300 CFU/g, 1.0 mL of the 1:10 initial suspension represented 0.1 g, and >30 colonies therefore corresponded to >300 CFU/g. Exact counts above this reporting category were not retained; all such values remain right-censored and have no numerical mean or standard deviation.

No.	Analytical category	Parameter	Sample 1 result	Sample 2 result	Sample 3 result	Sample 4 result	Sample 5 result	n < LOQ	Benchmark count 1	Benchmark count 2 / positive n	Comment
1	Goat cheese: Pogradeci	β-glucuronidase-positive *E. coli*	1900	1700	2000	1600	1800	0	Not applied	Not applied	Individual results are in CFU/g. The EU *Escherichia coli* m/M criterion for cheeses made from heat-treated milk or whey was not applied because this cheese was made from raw milk.
2	Goat cheese: Vlora	β-glucuronidase-positive *E. coli*	1000	1400	1200	1100	1300	0	Not applied	Not applied	Individual results are in CFU/g. The EU *Escherichia coli* m/M criterion for cheeses made from heat-treated milk or whey was not applied because this cheese was made from raw milk.
3	Goat cheese: Hasi	β-glucuronidase-positive *E. coli*	1200	1500	1800	1300	1700	0	Not applied	Not applied	Individual results are in CFU/g. The EU *Escherichia coli* m/M criterion for cheeses made from heat-treated milk or whey was not applied because this cheese was made from raw milk.

No.	Analytical category	Parameter	Sample 1 result	Sample 2 result	Sample 3 result	Sample 4 result	Sample 5 result	n < LOQ	Benchmark count 1	Benchmark count 2 / positive n	Comment
4	Goat cheese: Liqenasi	β-glucuronidase-positive *E. coli*	>300 CFU/g	>300 CFU/g	>300 CFU/g	>300 CFU/g	>300 CFU/g	0	5 above m; contextual	Above M: indeterminate	All five results were >300 CFU/g and therefore above m = 100 CFU/g. Because c = 2, the five-sample set would be unsatisfactory if the EU process-hygiene criterion were applicable at the sampled stage. The exact number of results above M = 1000 CFU/g cannot be determined from the right-censored values.
5	Sheep cheese: Bardhoke	β-glucuronidase-positive *E. coli*	>300 CFU/g	>300 CFU/g	>300 CFU/g	>300 CFU/g	>300 CFU/g	0	5 above m; contextual	Above M: indeterminate	All five results were >300 CFU/g and therefore above m = 100 CFU/g. Because c = 2, the five-sample set would be unsatisfactory if the EU process-hygiene criterion were applicable at the sampled stage. The exact number of results above M = 1000 CFU/g cannot be determined from the right-censored values.
6	Cow milk feta-type brined cheese	Beta-glucuronidase-positive *Escherichia coli*	<10	<10	<10	<10	<10	5	0 above m, contextual only	0 above M, contextual only	All five results were <10 CFU/g. The cheese was made from pasteurised milk and sampled after manufacture, but the exact stage of maximum expected *Escherichia coli* count was not established.

No.	Analytical category	Parameter	Sample 1 result	Sample 2 result	Sample 3 result	Sample 4 result	Sample 5 result	n < LOQ	Bench-mark count 1	Bench-mark count 2 / positive n	Comment
7	Goat yoghurt	Enterobacteriaceae	<10	<10	<10	<10	<10	5	N/A	N/A	All five results were <10 CFU/g; no product-specific m/M criterion was applied.
8	Sausage	Beta-glucuronidase-positive *Escherichia coli*	<10	<10	<10	<10	<10	5	N/A	N/A	All five results were <10 CFU/g; no product-specific m/M criterion was applied.
9	Pistachio cake	Enterobacteriaceae	<10	<10	<10	<10	<10	5	N/A	N/A	All five results were <10 CFU/g; no product-specific m/M criterion was applied.
10	Raw cow milk: Farm 1	Total aerobic colony count at 30 °C	3300000	3200000	3400000	3100000	3500000	0	5 above 100,000 CFU/mL	N/A	All values are CFU/mL; mean 6.52 ± 0.02 log CFU/mL. Formal assessment requires a rolling geometric average.
11	Raw cow milk: Farm 2	Total aerobic colony count at 30 °C	30000	33000	36000	31000	35000	0	0 above 100,000 CFU/mL	N/A	All values are CFU/mL; mean 4.52 ± 0.03 log CFU/mL. Formal assessment requires a rolling geometric average.
12	Raw cow milk: Farm 3	Total aerobic colony count at 30 °C	440000	420000	480000	460000	400000	0	5 above 100,000 CFU/mL	N/A	All values are CFU/mL; mean 5.64 ± 0.03 log CFU/mL. Formal assessment requires a rolling geometric average.

No.	Analytical category	Parameter	Sample 1 result	Sample 2 result	Sample 3 result	Sample 4 result	Sample 5 result	n < LOQ	Bench-mark count 1	Bench-mark count 2 / positive n	Comment
13	Frozen chicken fillet	Unconfirmed presumptive *Salmonella* spp. culture-screening finding	Unconfirmed presumptive finding in 10 g	Unconfirmed presumptive finding in 10 g	Unconfirmed presumptive finding in 10 g	Unconfirmed presumptive finding in 10 g	Unconfirmed presumptive finding in 10 g	N/A	N/A	5 findings	An unconfirmed presumptive *Salmonella* spp. culture-screening finding was obtained from the 10 g test portion of each of the five independent product lots. Complete confirmatory identification, serological agglutination, species-level identification, serotyping and molecular characterisation were not performed.
14	Pork fillet	Unconfirmed presumptive *Salmonella* spp. culture-screening finding	Unconfirmed presumptive finding in 25 g	Unconfirmed presumptive finding in 25 g	Unconfirmed presumptive finding in 25 g	Unconfirmed presumptive finding in 25 g	Unconfirmed presumptive finding in 25 g	N/A	N/A	5 findings	An unconfirmed presumptive *Salmonella* spp. culture-screening finding was obtained from the 25 g test portion of each of the five independent product lots. Complete confirmatory identification, serological agglutination, species-level identification, serotyping and molecular characterisation were not performed.
15	Grilled chicken fillet	*Listeria monocytogenes*	Not detected in 25 g	Not detected in 25 g	Not detected in 25 g	Not detected in 25 g	Not detected in 25 g	N/A	N/A	0 positive	*Listeria monocytogenes* was not detected in 25 g in any of the five samples.

No.	Analytical category	Parameter	Sample 1 result	Sample 2 result	Sample 3 result	Sample 4 result	Sample 5 result	n < LOQ	Bench-mark count 1	Bench-mark count 2 / positive n	Comment
16	Goat cheese: Liqenasi	Coagulase-positive staphylo-cocci	<10	<10	<10	<10	<10	5	N/A	N/A	All five results were <10 CFU/g. Results are reported as coagulase-positive staphylococci; enterotoxins were not analysed and formal criterion applicability depends on cheese category and manufacturing stage.
17	Goat cheese: Liqenasi	Coliforms	<10	<10	<10	<10	<10	5	N/A	N/A	All five coliform results were <10 CFU/g. Coliform counts were used as descriptive hygiene information only.
18	Goat cheese: Pogradeci	Coliforms	<10	<10	<10	<10	<10	5	N/A	N/A	All five coliform results were <10 CFU/g. Coliform counts were used as descriptive hygiene information only.
19	Goat cheese: Vlora	Coliforms	<10	<10	<10	<10	<10	5	N/A	N/A	All five coliform results were <10 CFU/g. Coliform counts were used as descriptive hygiene information only.
20	Goat cheese: Hasi	Coliforms	>300 CFU/g	>300 CFU/g	>300 CFU/g	>300 CFU/g	>300 CFU/g	0	N/A	N/A	All five coliform results were >300 CFU/g. These are right-censored values referring to 1 g of sample; the exact counts above 300 CFU/g were not quantified. Coliforms were used as descriptive hygiene indicators.

No.	Analytical category	Parameter	Sample 1 result	Sample 2 result	Sample 3 result	Sample 4 result	Sample 5 result	n < LOQ	Bench-mark count 1	Bench-mark count 2 / positive n	Comment
21	Goat cheese: Liqenasi	Total aerobic colony count at 30 °C	>300 CFU/g	>300 CFU/g	>300 CFU/g	>300 CFU/g	>300 CFU/g	0	N/A	N/A	All five total aerobic colony count results were >300 CFU/g. These are right-censored values referring to 1 g of sample; the exact counts above 300 CFU/g were not quantified.
22	Goat cheese: Pogradeci	Total aerobic colony count at 30 °C	>300 CFU/g	>300 CFU/g	>300 CFU/g	>300 CFU/g	>300 CFU/g	0	N/A	N/A	All five total aerobic colony count results were >300 CFU/g. These are right-censored values referring to 1 g of sample; the exact counts above 300 CFU/g were not quantified.
23	Goat cheese: Vlora	Total aerobic colony count at 30 °C	>300 CFU/g	>300 CFU/g	>300 CFU/g	>300 CFU/g	>300 CFU/g	0	N/A	N/A	All five total aerobic colony count results were >300 CFU/g. These are right-censored values referring to 1 g of sample; the exact counts above 300 CFU/g were not quantified.
24	Goat cheese: Hasi	Total aerobic colony count at 30 °C	>300 CFU/g	>300 CFU/g	>300 CFU/g	>300 CFU/g	>300 CFU/g	0	N/A	N/A	All five total aerobic colony count results were >300 CFU/g. These are right-censored values referring to 1 g of sample; the exact counts above 300 CFU/g were not quantified.

No.	Analytical category	Parameter	Sample 1 result	Sample 2 result	Sample 3 result	Sample 4 result	Sample 5 result	n < LOQ	Bench-mark count 1	Bench-mark count 2 / positive n	Comment
25	Sheep cheese: Bardhoke	Total aerobic colony count at 30 °C	>300 CFU/g	>300 CFU/g	>300 CFU/g	>300 CFU/g	>300 CFU/g	0	N/A	N/A	All five total aerobic colony count results were >300 CFU/g. These are right-censored values referring to 1 g of sample; the exact counts above 300 CFU/g were not quantified.
26	Goat cheese: Pogradeci	Coagulase-positive staphylococci	<10	<10	<10	<10	<10	5	N/A	N/A	All five results were <10 CFU/g. Results are reported as coagulase-positive staphylococci; enterotoxins were not analysed and formal criterion applicability depends on cheese category and manufacturing stage.
27	Goat cheese: Vlora	Coagulase-positive staphylococci	<10	<10	<10	<10	<10	5	N/A	N/A	All five results were <10 CFU/g. Results are reported as coagulase-positive staphylococci; enterotoxins were not analysed and formal criterion applicability depends on cheese category and manufacturing stage.
28	Goat cheese: Hasi	Coagulase-positive staphylococci	<10	<10	<10	<10	<10	5	N/A	N/A	All five results were <10 CFU/g. Results are reported as coagulase-positive staphylococci; enterotoxins were not analysed and formal criterion applicability depends on cheese category and manufacturing stage.

No.	Analytical category	Parameter	Sample 1 result	Sample 2 result	Sample 3 result	Sample 4 result	Sample 5 result	n < LOQ	Bench-mark count 1	Bench-mark count 2 / positive n	Comment
29	Sheep cheese: Bardhoke	Coagulase-positive staphylo-cocci	<10	<10	<10	<10	<10	5	N/A	N/A	All five results were <10 CFU/g. Results are reported as coagulase-positive staphylococci; enterotoxins were not analysed and formal criterion applicability depends on cheese category and manufacturing stage.

Table S7. Statistical analysis details and robustness checks. Conventional ANOVA was restricted to exact numerical results; datasets reported only as >300 CFU/g were treated as right-censored and were not entered into these analyses.

Comparison	Data included	Software	Assumption tests and outcomes	Primary test	Effect size and CI	Post hoc / robustness check	Interpretation
Raw-milk goat cheeses by origin, E. coli	Pogradeci, Vlora and Hasi, n = 5 independent product lots per sampled origin group; log10 CFU/g. Liqenasi and Bardhoke were excluded because all values were right-censored at >300 CFU/g.	Python 3.13.5; SciPy 1.17.0; statsmodels 0.14.6.	Residual Shapiro–Wilk W = 0.967, p = 0.817; Brown–Forsythe F(2,12) = 1.20, p = 0.334.	One-way ANOVA on log10 counts: F(2,12) = 11.43, p = 0.00167.	η^2 = 0.656; ω^2 = 0.582. Stratified bootstrap 95% CI for η^2: 0.533 to 0.875 (100,000 resamples; seed 20260623).	Tukey HSD: Pogradeci a, Hasi ab, Vlora b. Pogradeci vs Vlora p = 0.00121; Pogradeci vs Hasi p = 0.106; Hasi vs Vlora p = 0.0616. Welch F(2, 7.43) = 15.39, p = 0.00228; Kruskal-Wallis H = 8.88, p = 0.0118.	An origin effect was detected among the three raw-milk goat-cheese groups. Only Pogradeci and Vlora differed significantly in Tukey HSD. Because origin was not separated experimentally from producer, process or lot, no causal interpretation is made, and the result should not be generalised beyond the sampled product lots.
Raw cow milk farms, total aerobic colony count at 30 °C	Three anonymised farms, n = 5 independent bulk-milk lots per farm, each obtained in a separate collection event; log10 CFU/mL.	Python 3.13.5; SciPy 1.17.0; statsmodels 0.14.6.	Log10-scale residuals: Shapiro-Wilk W = 0.939, p = 0.369; Brown-Forsythe F(2,12) = 0.687, p = 0.522. On the raw scale, Shapiro-Wilk W = 0.870, p = 0.0333 and Brown-Forsythe F(2,12) = 8.11, p = 0.00591, supporting log10 transformation.	One-way ANOVA on log10 counts: F(2,12) = 5928.00, p = 1.07×10^{-18}.	η^2 = 0.99899; ω^2 = 0.99874. Stratified bootstrap 95% CI for η^2: 0.99875 to 0.99963 (100,000 resamples; seed 20260623).	Tukey: all pairwise contrasts p < 0.001; Farm 1 a, Farm 3 b, Farm 2 c. Welch F(2,7.60) = 6059.39, p = 6.64×10^{-13}; Kruskal–Wallis H = 12.50, p = 0.00193.	Large and consistent differences were observed among the three sampled farms, with Farm 1 > Farm 3 > Farm 2 on the log10 scale. This was a cross-sectional screening comparison of independent bulk-milk lots obtained in separate collection events, not a regulatory compliance assessment based on a rolling geometric average.

Statistical values were recalculated from the individual results using log10-transformed counts. The recalculation reproduced the ANOVA, Welch and Kruskal-Wallis statistics. Corrections concern the treatment of censored observations, reproducibility of bootstrap confidence intervals, and interpretation of the experimental units and sampling design.
